# A review on biogenic synthesis, applications and toxicity aspects of zinc oxide nanoparticles

**DOI:** 10.17179/excli2020-2842

**Published:** 2020-09-22

**Authors:** Rajat Sharma, Rajni Garg, Avnesh Kumari

**Affiliations:** 1 Department of Chemistry, Rayat Bahra University, Mohali, Punjab, 140301, India; 2 Biotechnology Division, CSIR-Institute of Himalayan Bioresource Technology, Palampur, H.P., 176061, India; 3 Academy of Scientific and Innovative Research (AcSIR), CSIR-Institute of Himalayan Bioresource Technology (CSIR-IHBT), Palampur, H.P., 176061, India

**Keywords:** nanotechnology, biogenic synthesis, nanoparticles, degradation, toxicity

## Abstract

Nanoparticles (NPs) have become an important field of research over the past several decades with applications in various sectors, such as biomedical, cosmetic, food and many others, because of their unique characteristics. The green synthesis of nanoparticles has been preferred because of the naturally occurring reductants present in biological systems that decreases exposure to toxic chemicals compared with physico-chemical methods and is eco-friendly. Zinc oxide (ZnO) NPs exhibit broad and potential applications in different fields with their specific characteristics such as surface area, size, shape, low toxicity, optical properties, high binding energy and large band gap. This paper focuses on the bio-synthesis of ZnO NPs by utilizing extracts of different plant parts (stem, flower, fruit, peel, and leaves) through efficient, economical, simple, pure, and eco-friendly green routes. In this process, zinc salts have been used as precursor and phytochemicals in the plant extract reduce the metal salt to lower oxidation state as well as stabilize the ZnO NPs. The morphological and physico-chemical properties of obtained NPs analyzed by various characterization techniques have been discoursed. Further, antimicrobial activity and potential photocatalytic application in terms of the degradation of dyes have also been reviewed in addition to the toxicity aspects of these NPs on human beings and animals.

## Introduction

**Figure 1:**
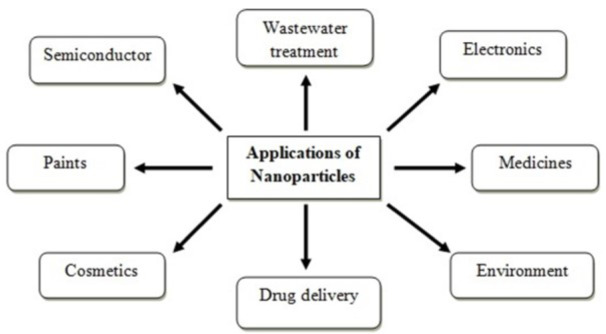
Applications of nanoparticles

Nanotechnology is an evolving field of science and technology that focuses on the synthesis and development of nanomaterials having particle size in between 1-100 nm (Rajiv et al., 2013[47]). Nanoscience and nanotechnology are an interdisciplinary field of study that has been found to embed the desirable impact on economy and society in the 21^st^ century and it has the potential for generating next industrial revolution. The nanomaterials such as nanocomposites (Bordbar et al., 2018[9]; Pandey et al., 2017[40]; Das and Srivasatava, 2016[11]), nanotubes (Eatemadi et al., 2014[15]; Rao et al., 2007[49]), nanoelectronics (Pês et al., 2014[42]; Park et al., 2016[41]), and NPs are widely used these days. NPs have drawn more attention of scientist community because of their unique physico-chemical properties. NPs are the microscopic particles having size ranging between 1-100 nm (10^-9^ m) which can't be seen by human eyes but can be measured by highly sophisticated instruments with high resolution (Ramesh et al., 2014[48]). When size of material reduces to micro and nano scale; their properties completely change as compared to bulk material. Due to change in properties of nanomaterials at nanoscale, these are widely used in various scientific and technological fields (Figure 1(Fig. 1)) (Sundaram et al., 2012[55]). 

Recently, the scientific community has shown interest to synthesize metal and metal oxides NPs (Ajmal and Saraswat, 2017[2]). Among these NPs; ZnO NPs exhibit most significance and wide range of applications including photocatalytic, antimicrobial and water purification. ZnO NPs exhibit different properties from the conventional zinc oxide particles (Sangeetha et al., 2011[51]). ZnO NPs have large band gap (3.37 eV) with ability to produce electron-hole effectively when UV radiations fall on their surface that allows their use in electronic devices and as photocatalyst for the degradation of textile waste and removal of industrial heavy metal ions. ZnO NPs have high binding energy (60 meV) at room temperature that allows their use in photo electronics, semiconductors, solar cells, surface acoustic wave devices and field emitters (Matinise et al., 2017[32]). These NPs are also used in cosmetic industries for the production of sunscreen lotions to protect the human body from the UV radiations (Osmond and Mccall, 2010[39]). The most significant properties of ZnO NPs such as no toxicity and biocompatibility make them desirable in specific biomedical applications (Suresh et al., 2018[60]; Malek and Nahid, 2018[31]; Jafarirad et al., 2016[21]).

**Figure 2:**
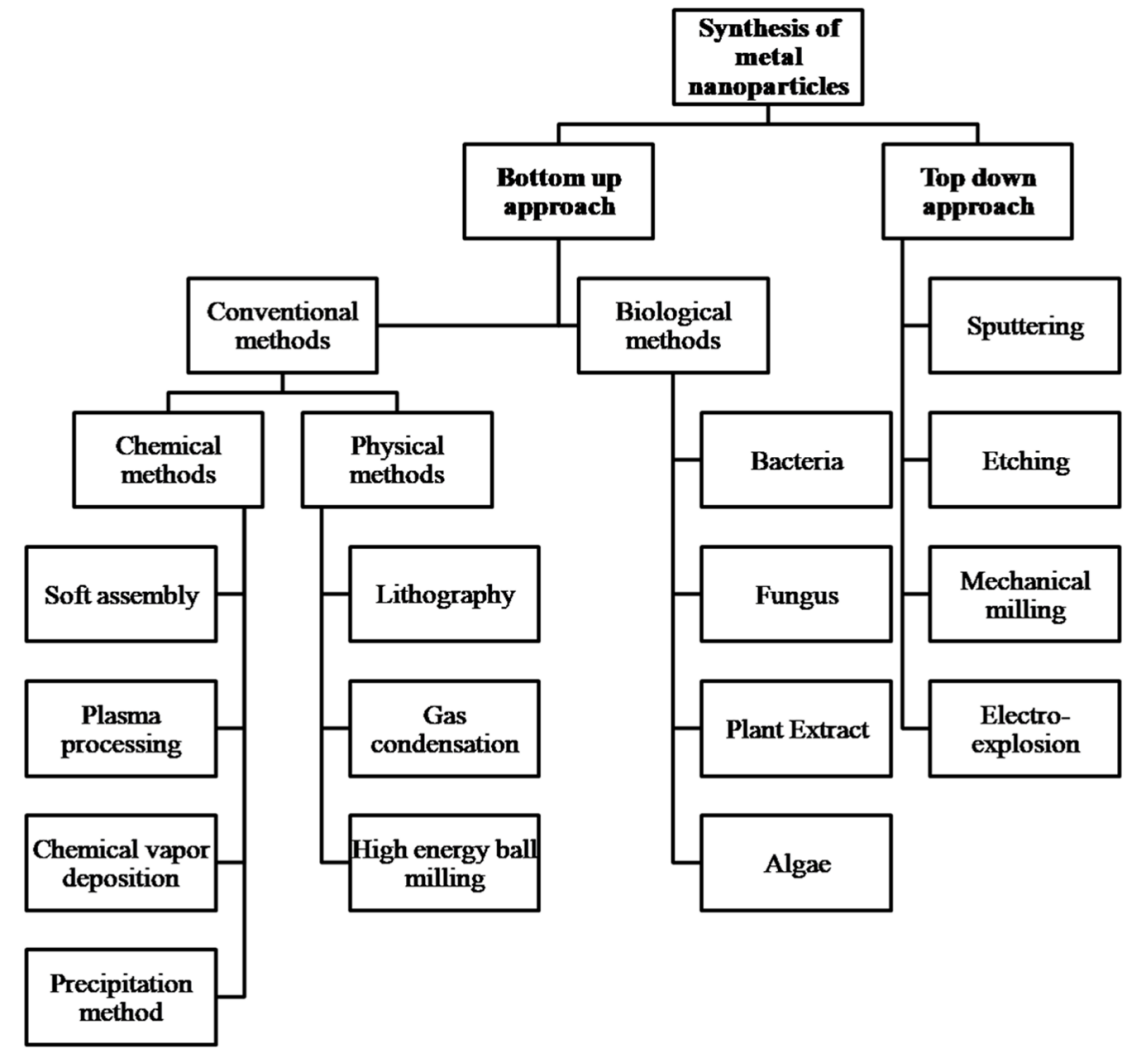
Synthesis of NPs by Bottom up and Top down approaches

Top Down and Bottom up approach are the two approaches from which NPs can be synthesized (Figure 2(Fig. 2)) (Priyadarshana et al., 2015[44]). Top down approach includes sputtering, etching, mechanical milling, and electro-explosion while bottom up approach includes two main methods from which the NPs are synthesized i.e. conventional methods namely; physical, chemical and biological methods (Hasnidawani et al., 2016[20]; Diwan et al., 2015[13]). The NPs synthesized by conventional methods are pure but at the same time, not cost effective and often lead to formation of toxic byproducts that may have adverse effects when used for medical applications. In addition, these methods require additional capping and stabilizing agents (Suntako, 2015[57]). This issue does not appear when NPs are synthesized by the green route that is a kind of bottom up approach resulting in oxidation/reduction reaction (Vijayakumar et al., 2018[64]). In green synthesis, extract of diverse parts of medicinal plants acts as the reducing medium and the constituent phytochemicals act as capping agent, biocatalyst and natural stabilizer for NPs (Senthilkumar and Sivakumar, 2014[53]). It doesn't make use of high energy, temperature, pressure, costly instruments and environmentally hazardous chemicals (Nagar and Nagar, 2015[37]). Thus, in contrast to the expensive and toxic methods, the green synthesis of NPs is less expensive, non-toxic and eco-friendly (Sundrarajan et al., 2015[56]; Lingaraju et al., 2016[29]).

## Biogenic Synthesis

ZnO NPs are synthesized by means of numerous conventional approaches; for instance: co-precipitation, sol-gel method, high energy ball milling, electrochemical and electrophoretic depositions, chemical vapor deposition, chemical vapor, and microwave-assisted combustion methods (Darroudi et al., 2013[10]; Azizi et al., 2017[7]). Nowadays, ZnO NPs are also synthesized by biogenic methods using plants, yeast, fungi, bacteria and algae (Santhoshkumar et al., 2017[52]). Although, various biological species such as microorganisms including fungi and bacteria can be used for green synthesis, yet plants are the most common biological substrate used for green synthesis of metal NPs (Table 1(Tab. 1); References in Table 1: Abdul Salam et al., 2014[1]; Ajmal and Saraswat, 2017[2]; Anbuvannan et al., 2015[4]; Azizi et al., 2016[6]; Bhuyan et al., 2015[8]; Davar et al., 2015[12]; Dobrucka and Długaszewska, 2016[14]; Fowsiya et al., 2016[16]; Fu and Fu, 2015[17]; Gandhi et al., 2017[18]; Hajinasiri et al., 2016[19]; Jafarirad et al., 2016[21]; Jamdagni et al., 2018[22]; Janaki et al., 2015[23]; Karnan and Selvakumar, 2016[25]; Kumar et al., 2014[28]; Lingaraju et al., 2016[29]; Madan et al., 2016[30]; Malek and Nahid, 2018[31]; Nagajyothi et al., 2013[34], 2014[36], 2015[35]; Nava et al., 2017[38]; Prachi et al., 2019[43]; Rajendran and Sengodan, 2017[46]; Rajiv et al., 2013[47]; Ramesh et al., 2014[48]; Sangeetha et al., 2011[51]; Santhoshkumar et al., 2017[52]; Senthilkumar and Sivakumar, 2014[53]; Sundrarajan et al., 2015[56]; Supraja et al., 2016[58]; Suresh et al., 2015[59], 2018[60]; Thatoi et al., 2016[61]; Thema et al., 2015[62]; Vennila and Jesurani, 2017[63]; Vijayakumar et al., 2018[64]; Zhao et al., 2015[65]; Zheng et al., 2015[66]) due to the reason that plants are more cost-effective, easier to handle and non-toxic (Suresh et al., 2015[59]; Azizi et al., 2016[6]). In addition, no safety issues are involved that are associated while dealing with microorganisms. During green synthesis of ZnO NPs from plant extract, the suitable parts such as stem, leaves, flowers, fruit etc. are washed with water and simple extraction techniques are used to obtain plant extracts. After filtration, elution and separation, the extract is either dried or used as such to react further with zinc precursor under different conditions of pH and temperature. The molar concentration of the extract and zinc precursor is varied to obtain good yield of the product (Thema et al., 2015[62]; Nagajyothi et al., 2015[35]; Janaki et al., 2015[23]).

These phytochemicals include various bio-active compounds such as polyphenols, flavonoids, and saponins that form chelate complex with the metal ion and also stabilize the NPs. The main mechanism responsible for the formation of ZnO NPs involves the simultaneous reduction and oxidation of cationic zinc ion by the phytochemicals present in the extracts. These phytochemicals not only act as reductants but also as the stabilizing and capping agents. In addition, no additional chemical stabilizers, are required. After the completion of the reaction, the product is subjected to annealing to obtain ZnO NPs (Zheng et al., 2015[66]). The ZnO NPs produced by means of plant extract (stem, root, leaves, flower, and peels) show higher photocatalytic, antimicrobial, and antioxidant properties as compared to the NPs synthesized by fungi, bacteria, algae, and yeast (Supraja et al., 2016[58]). This is due to the incorporation of phytochemicals such as carbohydrates, alkaloids, tannins, polyphenolic compounds, vitamins, proteins, amino acids, and terpenoids etc. present in different plant parts that act as capping agents during bio-synthesis of these NPs (Thatoi et al., 2016[61]; Dobrucka and Długaszewska, 2016[14]; Jamdagni et al., 2018[22]).

The green route was used to obtain ZnO NPs by using zinc nitrate as precursor and leaf extract obtained from *O. basilicum* *L. *which acted as active bio reducing and stabilizing agent. The nanoscale ZnO NPs with particle size of 14.28 nm and hexagonal shape were confirmed from XRD analysis (Abdul Salam et al., 2014[1]). ZnO NPs have been synthesized by green and environmentally-friendly route utilizing *M. charantia* leaf extract. The absorption maxima at 374 nm confirmed the formation of ZnO NPs. The GC-MS investigation of the *M. charantia* leaf extract revealed the presence of nonacosane and other phytochemicals viz phenols, and aliphatic amines that played an important role in reduction and stabilization processes. SEM analysis confirmed spherical NPs with size 12.82 nm (Gandhi et al., 2017[18]). 

Corn silk leaf extract was used to synthesize ZnO NPs in an easy, one step, economical, efficient and environmentally-friendly way. In this approach, the zinc salt solution was treated with the leaf extract at 84-90 ^o^C in absence and presence of 0.1 M NaOH solution that was used to maintain pH of the solution above 8. The SEM micrograph showed that the NPs synthesized by the first method have spherical shape (average size 16.69 nm) while nano rods and nano sheets have been obtained by the second method. The absorption peak at 355 nm was obtained during UV-Vis spectrophotometric analysis (Hajinasiri et al., 2016[19]). *P. trifoliata* fruit extract and zinc nitrate solution was used to obtain the stable ZnO NPs that have been used as catalyst for the condensation of acetophenone and 3,4-dimethyl benzaldehyde. The absorption peak was obtained at 327 nm in the UV-Vis absorption spectrum. TEM analysis study indicated the size of the NPs ranging from 8.48-32.51 nm (Nagajyothi et al., 2013[34]).

## Photocatalytic Activity of ZnO Nanoparticles

**Figure 3:**
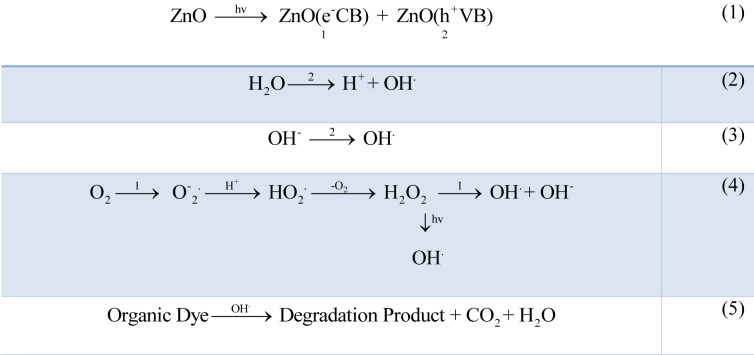
Mechanism for photocatalytic activity of ZnO NPs

**Figure 4:**
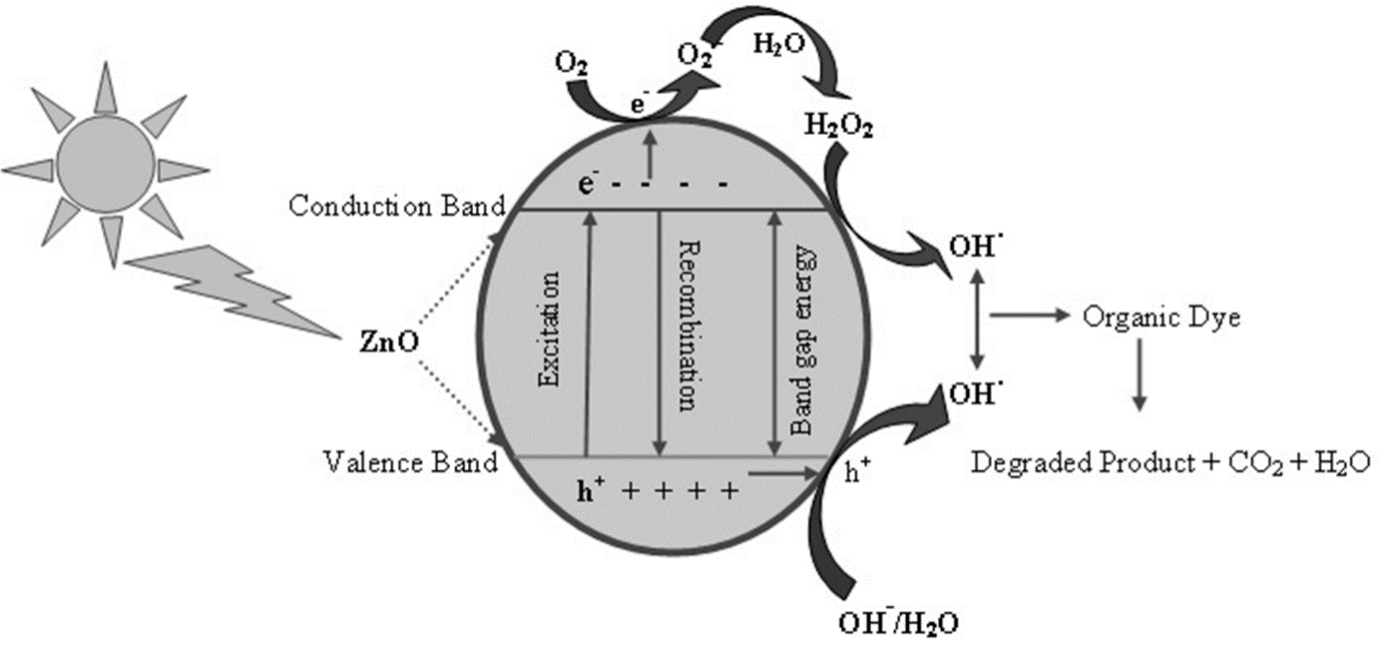
Schematic energy band diagram for photocatalytic activity of ZnO nanoparticles

Waste water as well as organic wastage from many industries such as textile industries, paint and dye industries contain non-biodegradable dyes that accumulate in ground water, and environment. The researchers and scientist community have been developing various new biodegradable methods which are simple, efficient, cost-effective, non-toxic and eco-friendly. Their photocatalytic activity is a function of size, shape, surface and optical activity. In this context, ZnO NPs have been synthesized by green route that show better photocatalytic activity (Fu and Fu, 2015[17]). The irradiation of ZnO NPs by sunlight having high photonic energy results in the excitation of electrons to empty conduction band producing electron-hole pairs which migrate to ZnO NPs surfaces and undergo oxidation/reduction where H^+^ reacts with water molecules and OH^-^ forming OH^.^ (hydroxyl radicals). The electrons upon reacting with oxygen produce superoxide free radical anions resulting in the formation of hydrogen peroxide that reacts further with superoxide radicals to form OH radicals. The resulting OH radicals are strong oxidizing agents that react with organic and inorganic pollutant absorbed on the surface of ZnO to produce intermediate compounds and convert to green compounds i.e. H_2_O, CO_2_ and inorganic compounds (Rauf and Ashraf, 2009[50]; Rajamanickam and Shanthi, 2016[45]) (Figure 3(Fig. 3)). The mechanism for photocatalytic activity of ZnO NPs can be represented as shown in Figure 3(Fig. 3) while the schematic energy band diagram has been represented in Figure 4(Fig. 4).

When a small quantity of ZnO NPs is added to dye solution, the rate of photodegradation of methylene blue dye is slow but on increasing the concentration, the rate of photodegradation also increases. This is due to size, structure, larger surface area over volume ratio and chemical properties of ZnO NPs (Kumar et al., 2014[28]). The solution is illuminated under the UV light without photocatalyst, and the amount of methyl orange dye degraded is observed. If the solution containing photocatalyst placed in the dark, there is no detectable change taking place indicating that there is necessity of both UV light and ZnO as a photocatalyst for the degradation of dyes (Vennila and Jesurani, 2017[63]).

Leaf extract of *P. amboinicus* was used as bioactive compounds for the preparation of pale yellow ZnO NPs. The absorption maxima at 375 nm was obtained during UV-Vis spectrophotometric analysis. SEM results revealed that the biosynthesized sample exhibited rod shape with size as 88 nm. The UV-Vis studies indicated band gap for ZnO NPs as 3.07 eV. The synthesized NPs showed good photodegradation activity against methyl red dye (Fu and Fu, 2015[17]). ZnO NPs have been synthesized using variable concentration of *S. grandiflora* leaf extract as an active bio-reductant for reduction of zinc nitrate used as a precursor material for production of NPs. The UV-Vis study showed the absorption at 235 nm indicating the formation of photosensitive ZnO NPs. SEM study indicated that ZnO NPs have spherical shape and the average particles size was 15-35 nm. The NPs showed potential as photocatalyst to remove the organic pollutant present in seafood industry during effluent treatment (Rajendran and Sengodan, 2017[46]).

ZnO NPs synthesized using *A. carnosus* exhibited strong photo-degradation activity as tested by using Heber multi photo reactor against methylene blue dye. On the addition of ZnO NPs catalyst to methylene blue dye, the color of methylene dye changed from blue to colorless showing the photodegradation and absorbance of dye in UV-Vis region (Anbuvannan et al., 2015[4]). *A. indica* (Neem) leaf extract synthesized ZnO NPs have hexagonal (Wurtzite) shape confirmed by PXRD pattern examination. The increasing concentration of extract resulted in the structural transformation of NPs. The obtained ZnO NPs degraded methylene blue and showed potential in photocatalysis (Madan et al., 2016[30]). A similar study reported photocatalytic potential of ZnO NPs synthesized from *A. indica* (Neem) extract was also explored. On addition of ZnO NPs as photocatalyst, the concentration of methylene blue dye decreased by 50 % after 30 minutes. At the end of the reaction (180 minutes), it was observed that methylene blue dye degraded from 82 % of its initial value (Bhuyan et al., 2015[8]). Lemon juice synthesized ZnO NPs have semispherical shape and an average particle size <50 nm. The particle size was further increased with increasing concentration of lemon juice extract. The XRD study found that the average size of crystalline NPs is approximately 20 nm as calculated by the Scherrer formula. These ZnO NPs showed good photocatalytic activity when used for decolorization and treatment of textile dyes (Davar et al., 2015[12]).

Another study synthesized microwave assisted ZnO NPs using *C. edulis* fruit extract by using microwave assisted extraction methods. XRD analysis revealed hexagonal crystalline structure of the NPs while, SEM showed flower shaped NPs with average size ranging between 50-55 nm. The photocatalytic degradation activity of prepared sample was tested towards organic pollutant, Congo red dye (Fowsiya et al., 2016[16]). 

An additional study, used stem extract of *R. cordifolia* for simple, low cost, economical and environmentally friendly synthesis of ZnO NPs from zinc nitrate solution. The obtained NPs were found to have spherical shape with average size 20.02 nm as confirmed by XRD and SEM-EDX analysis. ZnO oxide NPs showed good antioxidant properties and photocatalytic activity against methylene blue dye (Prachi et al., 2019[43]). ZnO NPs with band gap 3.37eV and 3.87 eV have been obtained using peel extract of* C. paradisi* (grape fruit). The UV-Vis absorption peak in the range of 360-375 nm was observed for ZnO NPs. The active compounds viz flavonoids, limonoids, and carotenoids molecules acted as bio-reductant in the synthesis of ZnO NPs. The DLS and TEM analysis revealed NPs with spherical shape and size ranging from 12-70 nm were indicated by TEM and results were found to coincide with DLS. The obtained NPs exhibited noteworthy 1,1-diphenyl-2-picrylhydrazyl scavenging activity, photocatalytic degradation activity against methylene blue dye and antioxidant activity (Kumar et al., 2014[28]). 

*Nephelium lappaceum *(Rambutan) fruit peel extract was used to obtain ZnO NPs*. *During the synthesis process, the phytochemicals present in the fruit extract acted as bio-reductant for the synthesis of NPs. The Scherrer equation was used to calculate the average size which was found to be 25.67 nm. The SEM micrograph revealed the spherical shape of NPs with diameter of 25-40 nm which was also matched with TEM micrographs. The degradation activity of dye was examined under illumination of UV light. ZnO NPs exhibited good photocatalytic activity during photodegradation of methyl orange (MO) dye under sunlight and showed around 83.99 % decolorization efficiency at 120 minutes of illumination (Karnan and Selvakumar, 2016[25]).

Fruits contain variety of phytochemicals that can be used as bio-reductant for the synthesis of NPs. A mixture of various fruit extracts was also used to synthesize ZnO NPs. Peels of *C. paradise* (grape fruit), *C. sinensis* (orange), *L. esculentum* (tomato) and *C. aurantifolia* (lemon) have been used to prepare extracts containing active bio-reductants such as flavonoids, limonoids and carotenoids that act as ligation agents as well as stabilizing and capping agents for NPs. The bio-inspired ZnO NPs exhibited polyhedral shapes with average size equal to 19.66 nm during TEM studies. The prepared NPs showed good photocatalytic and antibacterial activity. The photocatalytic degradation of methylene blue under UV illumination after 180 minutes showed that ZnO NPs synthesized using tomato extract exhibited maximum absorbance or photodegradation (97 %) due to small size (9.7 nm), larger surface area and highly optical properties as compared to ZnO NPs synthesized using lemon (96 %), and orange (77 %) (Nava et al., 2017[38]).

## Antimicrobial Activity of ZnO NPs

Biogenically synthesized ZnO NPs also have shown antimicrobial activity against wide range of microbes. In this section we have discussed antimicrobial activity of ZnO NPs. Most of the bacteria and pathogenic fungi are harmful for environment, agriculture, and living organisms. The antibacterial character of ZnO NPs against pathogenic fungi and bacteria is due to change in the cell permeability when the plasma membrane of bacterial cell comes in contact with ZnO NPs. This is due to the reason that ZnO NPs move to the cytoplasm and affect the normal functioning of cell resulting in the formation of zone of inhibition against the microbes. Further, ZnO NPs damage the cell membrane which results in the death of bacteria. This can be explained by the mechanism that oxygen species are released on the surface of NPs that react with hydrogen to produce hydrogen peroxide. The generated hydrogen peroxide either stops the growth of bacteria or kills the bacteria (Santhoshkumar et al., 2017[52]). The bacterial cell membrane disruption takes place by ZnO NPs, due to formation of superoxide and hydroxyl radicals. The zone of inhibition is directly proportional to the antibacterial activity of NPs, but inversely proportional to the size of ZnO NPs. Hence, as the size of NPs decreases, higher is the zone of inhibition and greater is the antibacterial action. The formation of hydrogen peroxide is related to the size and surface area of synthesized NPs. Smaller the ZnO NPs and larger the surface zone per unit area, greater is the formation of oxygen species and higher is the formation of hydrogen peroxide. The antibacterial activity has also been found to depend upon the shape of nanoparticles, type of synthesis and concentration of the ZnO NPs (Janaki et al., 2015[23]). 

ZnO NPs were obtained by using *P. caerulea* fresh leaf extract and zinc acetate solution. The peak at 380 nm indicated the formation of zinc oxide NPs by UV-Vis spectral analysis. The X-rays diffraction study revealed the size of synthesized NPs as 37.67 nm. The topography and roughness of NPs was analyzed by atomic force microscopy. SEM analysis determined the shape of *P. caerulea* capped ZnO as spherical with average diameter as 70 nm. These ZnO NPs were further tested for their antibacterial activity against urinary tract infecting Gram-positive bacterium, *Enterococcus* sp. and Gram-negative bacterium, *E. coli*. The results showed that ZnO NPs exhibited very good zone of inhibition against the pathogenic culture as compared with plant extract (Santhoshkumar et al., 2017[52]).

A pure, simple low cost and environmentally friendly approach was used for the synthesis of ZnO NPs by utilization bark extract of *B. ovalifoliolata* having active bioreductant compounds that played capping and stabilizing agents role. The UV-Vis analysis with localized surface Plasmon resonance absorption peak at 230 nm indicated the formation of fine powdered ZnO NPs. FT-IR study indicated that amines and proteins present in bark extract were involved in the formation of biologically highly stable round zinc oxide NPs. TEM study indicated the crystalline phase and morphology biosynthesized NPs. The average size of obtained powder sample was 20.3 nm. The antibacterial activity of prepared sample was tested by disk diffusion method against various cultured and uncultured fungal as well as bacterial species which were isolated from PVC pipes containing drinking water (Supraja et al., 2016[58]).

Zinc acetate solution was treated with *A. monophylla* leaf extract to obtain pale yellow colored ZnO NPs that were characterized by UV-Vis analysis and fluorescence spectrometry with peaks at 352 and 410 nm respectively. FTIR spectrum was utilized to recognize the particular phytochemicals viz tannin, organic acids, aromatic dicarboxylic acid etc. resulted for the reduction ions present in metal salts solution. The amide group present in proteins was supposed to play a crucial role as capping and balancing agents. The average size of obtained NPs was 33.07 as estimated by XRD analysis. The NPs were spherical in shape as confirmed by TEM as well as SEM image analysis. The results proved that ZnO NPs have better antimicrobial activity against many pathogens including *Pseudomonas aeruginosa*, *Klebsiella pnemoniae* than extract and standard drugs (Vijayakumar et al., 2018[64]).

Green tea has higher percentage of phytochemicals such as phenolic compounds, amino acids, proteins and lipids that help to stabilize the growth of NPs. ZnO NPs were obtained by using aqueous extract of *C. sinensis* (green tea) leaves mixed with the zinc acetate dehydrate at optimizing conditions. The UV-Vis spectral analysis exhibited a blue shift at 325 nm showing the development of ZnO NPs. These prepared NPs were analyzed by SEM which demonstrated the state of NPs as hexagonal with wurtzite structure and the normal size of NPs was 16 nm. The antibacterial properties were tested by Agar well-diffusion method against many pathogenic species, while the antifungal activity was tested against four fungal strains *Aspergillus fumigatus, A. flavus, Penicillium sp. *and* A. niger*. The results showed that biosynthesized NPs exhibit good sensitivity towards Gram-negative bacterium strain than Gram-negative strain and having good impact compared with synthetic drug (Senthilkumar and Sivakumar 2014[53]). 

The production of ZnO NPs from zinc sulfate was also carried out to explore the bioreductant potential of *A. barbadensis Miller* leaf extract. The XRD spectra displayed the ZnO NPs with 15 nm in size. FTIR spectral analysis showed the different types of active functional group present in the extract responsible for nucleation of ZnO NPs. ZnO NPs exhibited variable shapes including spherical, oval and hexagonal in the size range within 8-18 nm as confirmed by TEM. ZnO NPs showed extended spectrum with good antibacterial activity for *Beta lactamases* and *Pseudomonas aeruginosa *(Sangeetha et al., 2011[51]). 

ZnO NPs were synthesized by simple reaction between leaf extract of *A. carnosus* and zinc nitrate solution. The photoluminescence spectrum revealed a strong absorption peak below 400 nm of biosynthesized NPs. The band gap energy of zinc oxide NPs was calculated by using Kubelka-Munk plot equation. The result confirmed that as the concentration of extract increased, the ZnO NPs also exhibited greater band gap energy. FTIR spectrum indicated that the leaf extracts of *A. carnosus* have good percentage of polyphenols, carboxylic acids, polysaccharide, amino acids and proteins that played an important role in reduction, stabilization and capping of ZnO NPs. The structural analysis investigated by XRD revealed that the prepared NPs are crystalline with hexagonal wurtzite structure. The synthesized NPs exhibited significant antibacterial activities against human pathogens (Anbuvannan et al., 2015[4]).

*A. indica* (Neem) leaf extract was utilized as reducing medium for the synthesis of spherical ZnO NPs with average size ranging from 9.6-28.5 nm using zinc acetate. ZnO NPs were analyzed for their potential antibacterial and photocatalytic applications. The different convergence of ZnO NPs were used in shake-flask technique to test towards different bacterial strain i.e. *Staphylococcus aureus*, *Streptococcus pyrogenes* and *Escherichia coli *(Bhuyan et al., 2015[8]). 

Aqueous leaf extract of *C. pictus* was used to synthesize zinc oxide NPs using zinc nitrate. The hexagonal structure of zinc oxide NPs with size 29.11 nm was confirmed by XRD. The size and state of synthesized NPs were likewise investigated by TEM and SEM micrographs. The NPs exhibited good antibacterial potential against *B. subtili*, *S. paratyphi*, and* C. albicans*). The anticancer activity of zinc oxide NPs against mice cell lines was also studied. As the concentration of NPs was increased, the zone of inhibition as well as cell viability levels also increased (Suresh et al., 2018[60]). 

Active bio-components present in leaf extract of *P. hysterophorus* were used in presence of zinc nitrate as a precursor with the altering conditions of temperature and time to obtain white colored ZnO NPs with UV-Vis absorption peak at 374 nm. The presence of phytochemicals such as amine, 1,3-diketone, alkynes, secondary sulfonamide and phosphorous compound in leaf extract was characterized by FTIR study. ZnO NPs synthesized by using different concentrations of *Parthenium* leaf extract were found to be stable and having spherical, and hexagonal shapes as analyzed by SEM and TEM microscopic analysis. At higher concentration of reacting species, agglomeration was also observed. The XRD result revealed that average size of obtained ZnO NPs was varying in between 27-84 nm. The antifungal action of ZnO NPs was tested against plant contagious pathogens (*Aspergillus flavus* and *A. niger*) and the result demonstrated that ZnO NPs have great antifungal potential (Rajiv et al., 2013[47]). 

The zinc nitrate as precursor and leaf extract of *S. nigrum* plant was employed for synthesis of pale yellow colored ZnO NPs. The shape of NPs was found by TEM analysis as semi-spherical with an average width of 2 nm. ZnO NPs were found to exhibit potential applications in antimicrobial action against Gram-positive (*Staphylococcus aureus*) microorganisms just as therapeutic field (Ramesh et al., 2014[48]). Highly stable brown colored ZnO NPs were obtained by green technique utilizing natural fruit extract of the *Gooseberry* and salt precursor of zinc acetate. The presence of different functional groups in aqueous extract was confirmed by the FTIR analysis with strong absorption bands at 516 cm^-1^ and 432 cm^-1^ that were assigned to Zn-O bond. The crystalline structure with average size as 15 nm was analyzed by XRD analysis. The SEM images revealed spherical structure of the ZnO NPs (Vennila and Jesurani, 2017[63]). 

Methanolic extract of *P. domestica* fruit peels was used to synthesize the ZnO NPs using zinc nitrate as precursor. The FTIR spectrum revealed the presence of functional groups attached with NPs that had active role as stabilizing agents in the development of stable NPs. The antibacterial properties of NPs were tested by analysis of zone of inhibition while antioxidant activity was tested by DPPH and H_2_O_2_ scavenging assay. The result indicated that ZnO NPs had good antioxidant potential and antibacterial action towards *E. coli*, *S. aureus*, *Pseudomonas aeruginosa *and *B. substilis *(Ajmal and Saraswat, 2017[2]). Optimized conditions were used to synthesize zinc oxide NPs by using zinc nitrate and aqueous extract of *P. avium *(cherries). The peak at 378 nm showed the formation of ZnO NPs as analyzed by UV-Vis analysis. The SEM images revealed spherical morphology of the prepared sample. The size of NPs was ranging from 6.5-20.18 nm as confirmed by XRD spectrum (Malek and Nahid, 2018[31]). 

The active bioreductants in the flesh extract of *R. canina* fruit were used as reducing agents for the zinc nitrate precursor. The antibacterial action of ZnO NPs was analyzed in terms of concentration against various bacterial growth. In general, on increasing the concentration of prepared NPs sample, the rate of zone of inhibition also increases as found against *Salmonella typhimurium*. The prepared NPs were found to have wide range of potential for various medical, clinical and industrial applications (Jafarirad et al., 2016[21]). 

Stem extract of *R. graveolens* containing active bioreductants was used for the formation of sable ZnO NPs by zinc nitrate precursor. The absorption band at 335 nm was investigated by UV-Vis absorption spectrum. The particles were found with crystalline size about 28 nm and were analyzed by SEM analysis. The hexagonal phase with quartzite structure of ZnO NPs was confirmed by PXRD. ZnO NPs showed good antioxidant and antimicrobial activities against *Klebsiella aerogenes *and* Pseudomonas desmolyticum *(Lingaraju et al., 2016[29]). 

Flower extract of *T. pratense* having phytochemicals such as carotene, vitamin C, phenolic acids and anthocyanins has also been used to reduce the salt solution. ZnO NPs exhibited good antibacterial activity as the inhibition zone against *E. coli*, *S. aureus* and *P. aeruginosa* that increased with increasing the concentration of ZnO NPs (Dobrucka and Długaszewska, 2016[14]). Zinc oxide NPs were synthesized from zinc acetate solution by an eco-friendly approach by using aqueous flower extract of *N. arbortristis *plant. TEM study showed that NPs obtained by altering conditions were having size ranging between 12-32 nm and hexagonal phase was confirmed by XRD spectrum. The antifungal activity of sample was tested against phytopathogens (Jamdagni et al., 2018[22]). 

Flower extract of *A. italica* and the salt solution of zinc acetate has also been used in the biosynthesis of ZnO NPs with significant antimicrobial properties. The XRD investigation demonstrated the crystalline structure and size of the biosynthesized ZnO NPs in the scope of 60-70 nm. The outcomes in TEM analysis indicated the acquired NPs were hexagonal with normal size ranging of 8-14 nm (Azizi et al., 2016[6]). Biocomponents of dry powdered extract of dry *Z. officinale *rhizome (Ginger) were used to obtain ZnO NPs from zinc carbonate by a simple and eco-friendly method with size in the range of 23-26 nm. The antimicrobial action of ZnO NPs was tested by well diffusion method and area of zone of inhibition indicating prevention of the growth of pathogenic organisms like *Penicillium notatum, Klebsiella pheumonia*, *S. aureus* and *Candida albicans *(Janaki et al., 2015[23]). 

The root extract of *P. trifoliata *was used for the synthesis of highly stable and one pot ZnO NPs by use of green bottom up approach by utilizing zinc nitrate solution. TEM picture confirmed that the obtained test sample was round in shape with particle average size range of 33.03-73.48 nm. The biosynthesized ZnO NPs showed good antioxidant and anti-inflammatory properties (Nagajyothi et al., 2015[35]). *C. rhizoma* root extract has also been used to synthesize ZnO NPs with good antibacterial activity against bacterial strain of *Bacillus megaterium*, *B. pumilus*, *B. cereus*, and *Escherichia coli *(Nagajyothi et al., 2014[36]). 

## Toxicity Issues of Green Synthesized NPs

Zinc oxide belongs to the most commonly used nanomaterial community (Santhoshkumar et al., 2017[52]). As a well-known photocatalyst, the degradation of environmental contaminants has earned much attention from researchers (Rajamanickam and Shanthi, 2016[45]). Zinc salts has long been used as active ingredient in lubricants (Arfat et al., 2017[5]), and emollients by the pharmaceutical industry (Mirzaei and Darroudi, 2017[33]). ZnO NPs containing drugs are commonly used as wound remedies, anti-infection medical products and disinfectants. ZnO NPs exhibit extensive applications in cosmetics, formulations for hair and skin care, protective sunshades, food additives, vitamins, etc. (Osmond and Mccall, 2010[39]). The most important use as antibacterial agents in ointments, lotions, body washings and surface coatings for preventing microorganism growth is well known (Suresh et al., 2015[59]). ZnO NPs have also been used to help people and livestock as a dietary supplement for stimulating body response against inflammation and to boost the immune system (Singh et al., 2017[54]). The increasing use of ZnO NPs in consumables and in the pharmaceutical sector has aroused the need for the investigation of the possible toxic effects of ZnO NPs on human health (Osmond and Mccall, 2010[39]). The advantages must be carefully weighed against possible adverse consequences as with other nanoparticles.

The most recorded deleterious consequences of ZnO NPs inhalation in the literature are toxic effects on human lungs (Osmond and Mccall, 2010[39]). The magnitude of the inflammatory disease caused by the exposure to ZnO NPs has been associated with their size and surface area (Kalpana and Devi Rajeswari, 2018[24]). Earlier studies report that as compared to dissolved zinc ions, ZnO NPs can induce severe inflammatory response (Alghsham et al., 2019[3]). A series of experiments on human red blood cells and white blood cells have been conducted to evaluate the cytotoxic properties of ZnO NPs. The concentration higher than 50 ppm has been observed to have cytotoxic effect that is attributed to increase in oxidative stress (Khan et al., 2015[27]). The available data shows clearly that ZnO NPs can also cause acute effects on fish, at concentrations higher than expected in the environment (Keerthana and Kumar, 2020[26]). In order to better understand the therapeutic benefits and avoid unintended cytotoxic effects and clinical diagnostic potential, a detailed review is therefore needed of ZnO NPs features, route of administration, target cells and related physiological processes. The long-term consequences still have to be studied for better and safe usage of these NPs.

## Conclusion

In conclusion green synthesis of ZnO NPs is much more safer and environment friendly than physical and chemical methods. Phytochemicals present in the plant extracts act as stabilizing and reducing agents for the ZnO NPs. ZnO NPs find application as antimicrobial agent and photocatalyst. The plant-based ZnO NPs can become a major field of research and can be used extensively in the food, pharmaceuticals and cosmetic industries. The potential applications of ZnO NPs as a photocatalyst, as well as the associated mechanism has also been discussed. ZnO NPs with unique characteristics and photocatalytic potential can prove a boon for degradation of harmful dyes and other chemicals present in water. Hence, the significant photocatalytic potential of these NPs can be tailored further for potential use in waste water treatment and heavy metal ion removal. However, the considerable lack of knowledge as to whether and in what form these particles are released into the atmosphere is a major challenge based on current toxicity testing. Further work should be carried out to investigate the behavior and fate of parts with changes in receiving media. This is critical for ecotoxicological studies which are of environmental relevance.

## Conflict of interest

The authors declare no conflict of interest regarding the publication of this manuscript.

## Figures and Tables

**Table 1:**
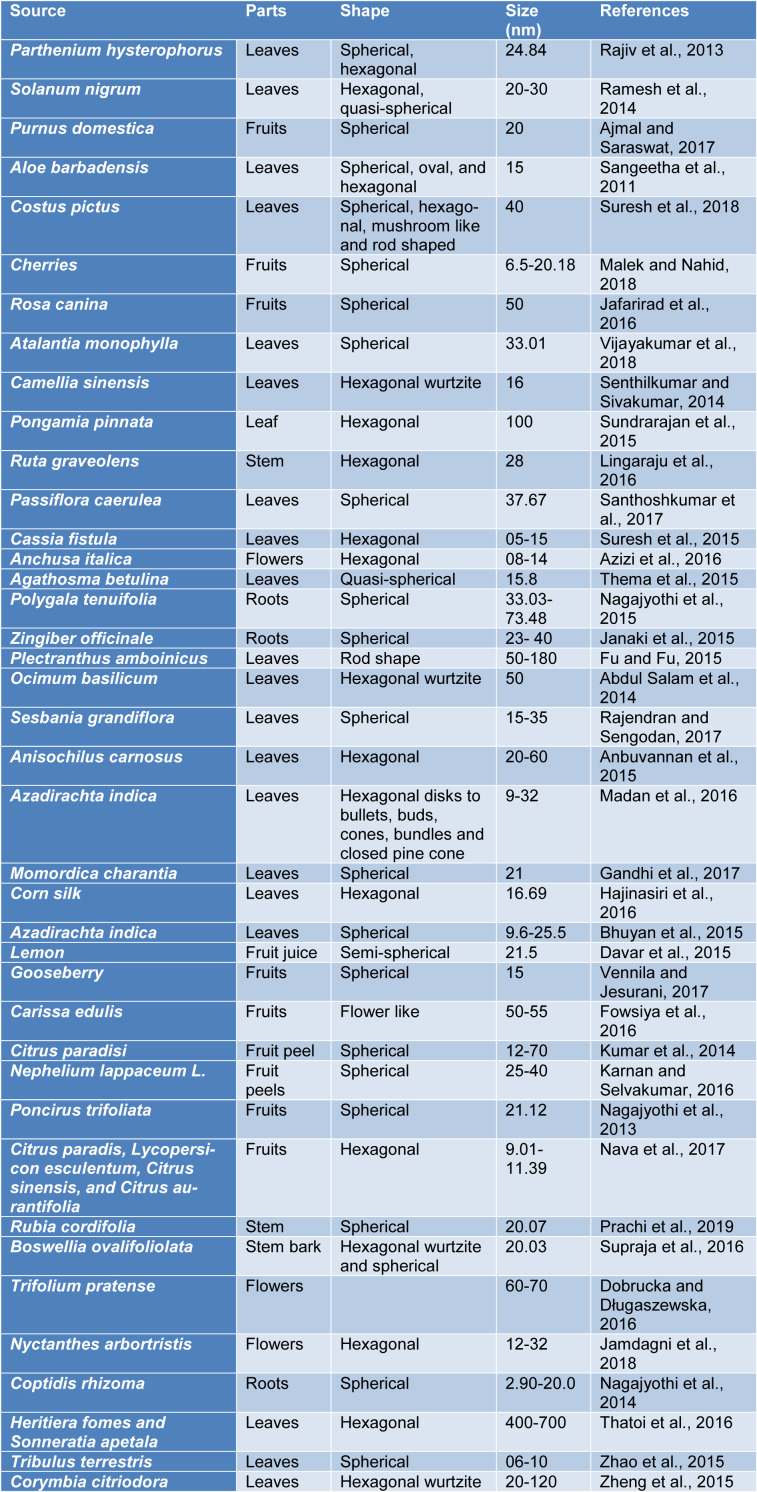
Biogenic synthesis of ZnO NPs by means of plant extract
